# Progression of Sclerouveitis to Endogenous *Fusarium* Endophthalmitis

**DOI:** 10.1155/2024/5549818

**Published:** 2024-08-20

**Authors:** Sairi Zhang, J. Anthony Chacko, Riley N. Sanders, Eric R. Rosenbaum, Philip W. Dockery, Ahmed B. Sallam

**Affiliations:** ^1^ College of Medicine University of Arkansas for Medical Sciences (UAMS), Little Rock, Arkansas, USA; ^2^ Department of Ophthalmology Jones Eye Institute University of Arkansas for Medical Sciences (UAMS), Little Rock, Arkansas, USA; ^3^ Department of Pathology University of Arkansas for Medical Sciences (UAMS), Little Rock, Arkansas, USA

## Abstract

**Introduction:** We describe a unique case of sclerouveitis that progressed to endogenous *Fusarium* endophthalmitis in a 69-year-old male with chronic lymphocytic leukemia (CLL). We highlight the risk of treating sclerouveitis with oral corticosteroids, which can exacerbate an infection and contribute to disease progression.

**Case Presentation:** A 69-year-old male with CLL on zanubrutinib, a second-generation Bruton's tyrosine kinase inhibitor, was admitted to the hospital for osteomyelitis of the left foot. At presentation, the patient also reported right eye pain for 1 week and vision loss over the course of 1 month. Vision in the right eye was hand motion. Slit lamp examination revealed scleral inflammation in the right eye with violaceous injection, chemosis, inflammation in the anterior chamber, and diffuse subconjunctival hemorrhage. There was significant corneal edema preventing fundus examination. B-scan ultrasonography demonstrated a flat retina with no vitritis or scleral thickening. Forty-eight hours after treatment with oral and topical corticosteroids, the patient's eye pain improved but his vision worsened. Repeat B-scan showed new-onset vitritis. Fungal culture obtained by diagnostic pars plana vitrectomy (PPV) revealed growth of *Fusarium*. The patient was treated with oral and intravitreal voriconazole in addition to intravenous voriconazole and amphotericin B for systemic therapy. Corticosteroids were discontinued. Despite aggressive therapy, the patient's disposition declined to the point of transitioning to comfort-focused care, and he passed away.

**Conclusion:** Endogenous fungal endophthalmitis is most commonly seen in immunocompromised patients, and oral corticosteroid therapy for such patients should be used with caution as it can worsen an infection. In cases of fusarial endophthalmitis, visual prognosis is poor.

## 1. Introduction

Endophthalmitis is a severe eye infection that can lead to irreversible vision loss and can arise from exogenous or endogenous sources. Exogenous endophthalmitis occurs following trauma or intraocular procedures, while endogenous endophthalmitis occurs with hematogenous spread of infectious organisms [1]. Among the fungal causes of endogenous endophthalmitis, *Candida* is the most common, while *Aspergillus* and *Fusarium* are rarer causes seen primarily in immunocompromised patients [2]. Factors contributing to suppressed immune states that increase the risk of fungal endophthalmitis include early or advanced age, malignancy, diabetes mellitus, and use of corticosteroids or other immunosuppressive therapies [2]. Diagnosis can be challenging due to clinical similarities to other conditions such as ocular syphilis, sympathetic ophthalmia, intraocular malignancy, and uveitis [3]. Thus, endophthalmitis should be included as a differential diagnosis for inflammatory ocular presentations. In this report, we demonstrate this by describing a case of sclerouveitis that progressed to endogenous *Fusarium* endophthalmitis in an immunosuppressed patient.

## 2. Case Presentation

**Figure 1:**
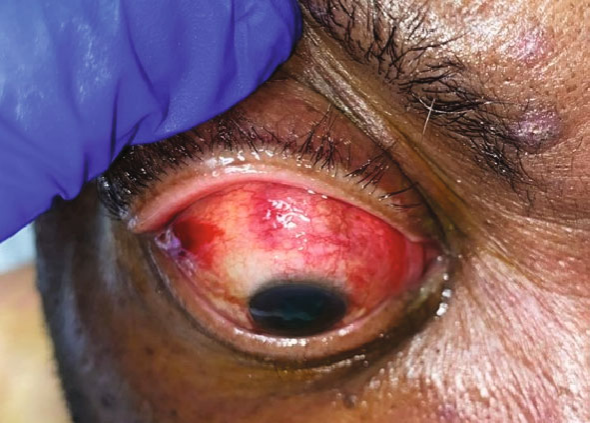
Clinical photograph with manual elevation of upper eyelid; mild right eyelid edema, diffuse subconjunctival hemorrhage with violaceous hue superiorly, 1+ chemosis, and corneal edema.

A 69-year-old male with chronic lymphocytic leukemia (CLL) presented to the emergency department with a nonhealing left foot wound, 1-week history of right eye (OD) pain, and gradual vision loss OD over 1 month. At presentation, he was receiving zanubrutinib, a second-generation Bruton's tyrosine kinase inhibitor, for CLL. Past medical history was significant for type II diabetes and recent left toe amputation. Ocular history was significant for diabetic retinopathy and cataract. The patient denied recent eye discharge or ocular trauma. He was afebrile with stable vital signs. White blood cell count was elevated at 23.1 K/*μ*L (ref. 3.6–9.5 K/*μ*L) with lymphocytosis, while absolute neutrophil count was decreased at 1.1 K/*μ*L (ref. 1.4–6.0 K/*μ*L). He was thrombocytopenic at 57 K/*μ*L (ref. 150–450 K/*μ*L). Erythrocyte sedimentation rate and C-reactive protein were elevated. The patient was admitted to the hospital for surgical management of osteomyelitis involving the left foot. Zanubrutinib was held due to acute infection. He received antibiotic therapy with doxycycline, levofloxacin, and metronidazole, though blood cultures were ultimately finalized as negative for growth. Ophthalmologic examination revealed that vision was hand motion OD and 20/70 in the left eye (OS). The right globe was tender to palpation. Intraocular pressures were normal in both eyes (OU). There was no afferent pupillary defect OU. Extraocular movements were full OU, and confrontational visual fields were full OS. Slit lamp examination revealed scleral inflammation OD with violaceous injection superiorly, chemosis, inflammation in the anterior chamber, and diffuse subconjunctival hemorrhage (Figure 1). There was also significant corneal edema preventing fundus examination. However, B-scan ultrasonography demonstrated a flat retina OD with no vitritis or scleral thickening.

The differential diagnosis included sclerouveitis versus ocular ischemic syndrome versus endogenous endophthalmitis. While hospitalized, the patient continued oral antibiotics for osteomyelitis of the left foot. Results of additional infectious disease laboratory testing were negative for HIV 1/2 antibodies, *T. pallidum* antibodies, *Toxoplasma* antibodies, and tuberculosis by T-SPOT® (Oxford Immunotec USA, Inc., Marlborough, MA). Autoimmune laboratory tests were also negative. Computed tomography (CT) of the orbits with and without contrast was unremarkable except for mild bilateral proptosis. Carotid duplex was performed with only mild disease of the bilateral internal carotid arteries. Due to the clinical examination findings and negative infectious work-up, the patient was started on oral prednisone 80 mg daily and 1% topical prednisolone acetate four times daily OD for treatment of sclerouveitis. After 48 h of treatment, the patient reported decreased right eye pain, but his vision worsened to light perception OD. Eye exam was unchanged; however, repeat B-scan OD revealed vitritis with a focal tuft of inflammation near the posterior pole.

**Figure 2:**
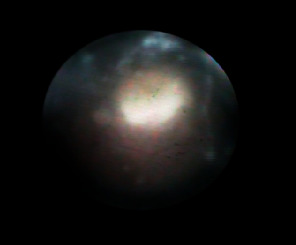
Intraoperative photo of large white fluffy retinal lesion.

**Figure 3:**
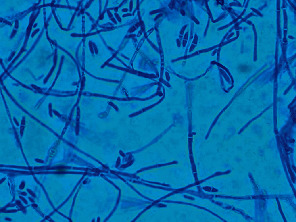
Lactophenol cotton blue preparation of vitreous biopsy fungal culture, × 1000 magnification: *Fusarium* sp. with characteristic sickle-form, “canoe-like” septate macroconidia.

Due to worsening vision OD and vitritis noted on B-scan ultrasonography, a diagnostic pars plana vitrectomy (PPV) with vitreous biopsy was performed for further investigation. In the operating room, vitritis was noted with a 19-gauge straight endoscope. There was a large infiltrate in the macula with areas of bleeding and small white retinal infiltrates (Figure 2). Additionally, the crystalline lens was found to be dislocated into the vitreous cavity. However, a lensectomy could not be attempted due to difficult visualization and active endophthalmitis. The vitreous specimen was sent for Gram stain and bacterial and fungal cultures. Due to the intraoperative findings, oral prednisone was discontinued and empiric oral voriconazole therapy was initiated in addition to the patient's current antibiotic regimen. As a result of the fungal culture growing *Fusarium* species at 48 h (Figure 3), an intravitreal voriconazole injection was administered OD following the PPV and again 4 days later. In addition, intravenous voriconazole and amphotericin B were initiated as therapy for systemic fusariosis.

Despite aggressive intravitreal and systemic antifungal therapy, the patient's disease progressed rapidly to septic shock and respiratory failure. He was transitioned to comfort-focused care and passed away.

## 3. Discussion

Given the severity of the disease, endophthalmitis must be promptly diagnosed and treated to save vision in the infected eye. However, there are many ocular conditions with similar presentations to that of endophthalmitis which complicate diagnosis and management. Noninfectious and infectious etiologies must be discerned. For our patient, noninfectious sclerouveitis was diagnosed after the initial infectious work-up came back negative. Sclerouveitis is typically associated with autoimmune disease but can be caused by an infectious etiology [4]. While corticosteroids are often used to treat sclerouveitis, they can exacerbate an infection. In some cases, however, steroids may elicit a good response in the initial stages [5]. In the patient's case, oral and topical steroids were started once sclerouveitis was suspected based on clinical examination. The patient's eye pain responded to the steroids, but his visual acuity continued to worsen. In retrospect, in the context of inflammation in an immunosuppressed host, systemic prednisone should have been held until further infectious work-up was completed.

To help diagnose fungal endophthalmitis, Priluck, Huang, and Breazzano identified that a more indolent course with a longer duration of pain and subjective visual acuity decline is more predictive of fungal endophthalmitis [3]. In addition, risk factors that should increase suspicion of fungal endophthalmitis include intravenous drug use, presence of an indwelling line, recent sepsis, hepatitis C, total parental nutrition use, and immunosuppressed state [3]. Immunosuppression was a significant risk factor for fungal endophthalmitis in the patient, for he had CLL and was on zanubrutinib therapy, which can cause neutropenia [6]. Other risk factors included his advanced age and diabetes mellitus.

Endophthalmitis caused by *Fusarium* species is most commonly seen in patients with hematologic malignancies and may be a clinical manifestation of disseminated fungal disease [7, 8]. Treatment for fungal endophthalmitis includes systemic antifungal therapy, intravitreal antifungal injection, and vitrectomy [9]. *Fusarium* is notable for its destructive effect on vision and high rates of resistance against many antifungal agents including fluconazole, itraconazole, and ketoconazole [10]. Voriconazole is a broad-spectrum antifungal approved for invasive fungal infections including *Fusarium*, with several studies suggesting safe and effective use with good ocular penetration [10, 11]. Ultimately, visual outcomes in cases of *Fusarium* endophthalmitis are poor even with appropriate antifungal therapy [12, 13].

As highlighted in this case report, sclerouveitis can progress to endophthalmitis following treatment with corticosteroids. If sclerouveitis is suspected, it is important to rule out infectious etiologies before beginning anti-inflammatory treatment with oral corticosteroids to avoid worsening of the infection. There should be a high degree of suspicion for fungal endophthalmitis in patients with immunocompromised states. It should be noted that endophthalmitis caused by *Fusarium* is associated with worse visual acuity outcomes.

## Data Availability

Data sharing is not applicable to this article as no new data were created or analyzed in this study.
